
JOURNAL INFORMATION
==============================
NLM Title Abbreviation: ScientificWorldJournal
Journal ID: ScientificWorldJournal
NLM Journal ID: 101131163
Journal ID: TSWJ

The Scientific World Journal

ISSN: 2356-6140
EISSN: 1537-744X
Publisher: Wiley

ARTICLE INFORMATION
==============================
PMCID: PMC5763829
PMID: 20694449
DOI: 10.1100/tsw.2010.142
Article ID: 870521
Article version: 1
Subjects: Clinical Images

Liver Abscess due to Sewing Needle Perforation

Jutte Ewoud H. *
Cense Huib A.
Department of Surgery, Red Cross Hospital, Beverwijk, The Netherlands
*Ewoud H. Jutte: ejutte@rkz.nlAcademic Editor: Daniel Batlle

Publication date: 2010
Electronic publication date: 2010 Aug 3
Volume: 10
First page: 1532
Last page: 1534
Received 2010 May 31; Revised 2010 Jun 16; Accepted 2010 Jun 17
Copyright: Copyright © 2010 Ewoud H. Jutte and Huib A. Cense.
Copyright year: 2010
License: This is an open access article distributed under the Creative Commons Attribution License, which permits unrestricted use, distribution, and reproduction in any medium, provided the original work is properly cited.
License URL: https://creativecommons.org/licenses/by/3.0/

Keywords: sewing needle perforation, liver abscess

==============================
Clinical Image
*TheScientificWorldJOURNAL* (2010) 10, 1532–1534
ISSN 1537-744X; DOI 10.1100/tsw.2010.142

TheScientificWorldJOURNAL
www.thescientificworld.com

### Liver Abscess due to Sewing Needle Perforation

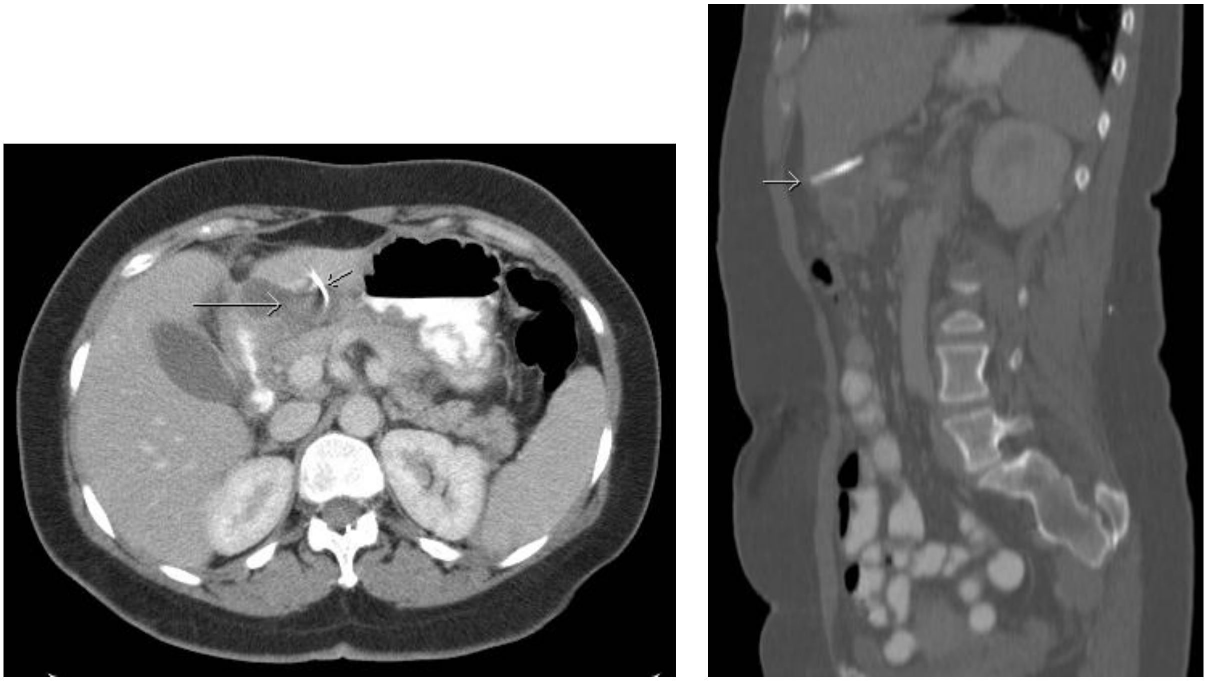

A B

**FIGURE 1.** (A,B) Abdominal CT scan showing a foreign body perforating through the duodenum in the left liver lobe (small arrows) and a liver abscess (big arrow).

Ewoud H. Jutte* and Huib A. Cense
*Department of Surgery, Red Cross Hospital, Beverwijk, The Netherlands*

**E-mail**: ejutte@rkz.nl

*Received May 31, 2010; Revised June 16, 2010; Accepted June 17, 2010; Published August 3, 2010*

**KEYWORDS**: sewing needle perforation, liver abscess

A 45-year-old female was admitted to the hospital with a 1-week history of right upper abdominal pain and nausea. Ultrasonography showed a thickened duodenum with infiltration. Subsequent gastroscopy did not reveal any abnormalities. CT scan showed a foreign body perforation at the duodenum and a liver

*Corresponding author.
©2010 with author.
Published by TheScientificWorld; www.thescientificworld.com abscess (Fig. 1A,B). A diagnostic laparoscopy was performed. During the operation, the abscess was drained (Fig. 2A,B) and a sewing needle was removed (Fig. 3A,B). The patient claimed to have no recollection of swallowing the needle and made a quick recovery.

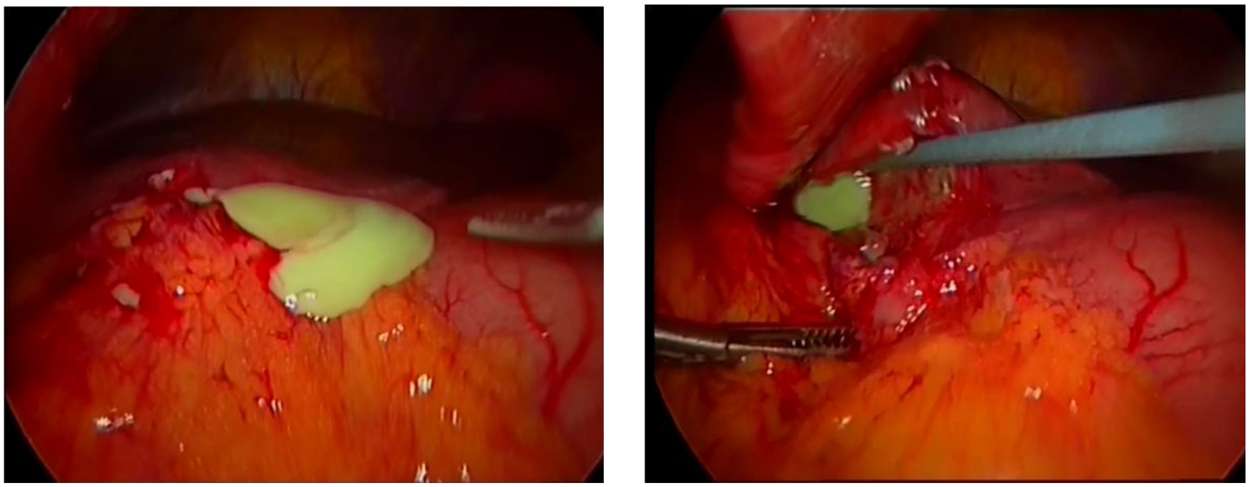

**A** **B**

**FIGURE 2.** (A,B) Laparoscopic imaging showing the liver abscess, which was drained by a suction device.

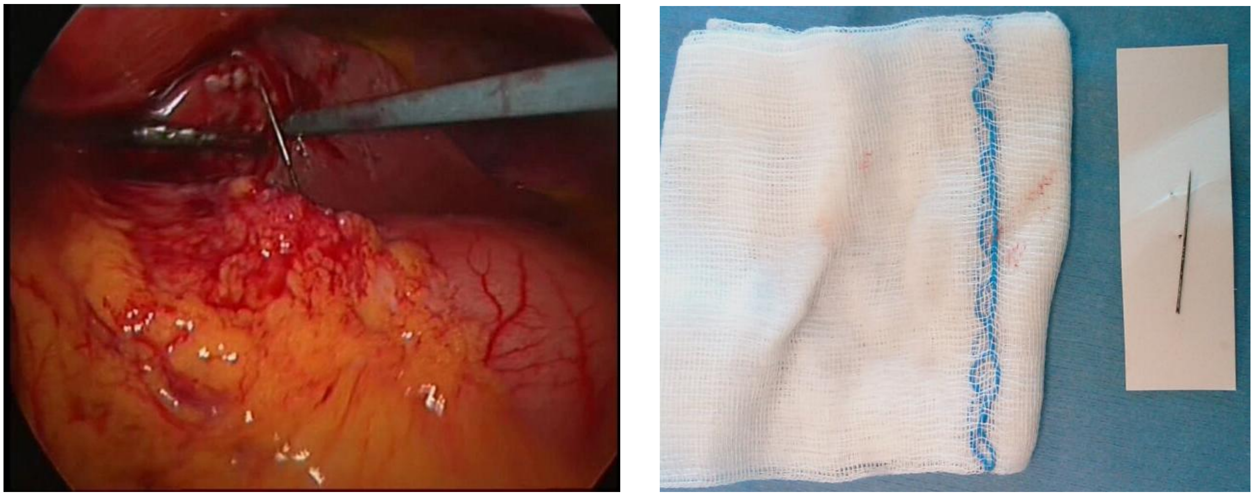

**A** **B**

**FIGURE 3.** (A) Laparoscopic imaging showing the perforating sewing needle. (B) Postoperative image of the removed sewing needle, lying beside a 10- × 10-cm gauge.

Although rare, gastrointestinal perforations due to ingested foreign bodies most frequently occur at ileocecal, rectosigmoid, and duodenal regions[1]. Sewing needle perforations into the liver have been reported in children, adults, and psychiatric cases. Symptoms can vary from mild gastric pain to signs of peritonitis[2]. “Wait and see” is recommended for asymptomatic patients with no complications. Symptomatic patients (e.g., liver abscess) need surgical intervention[1,2,3].

### REFERENCES

1. Bulakci, M., Agayev, A., Yanar, F., Sharifov, R., Taviloglu, K., and Ucar, A. (2009) Final destination of an ingested needle: the liver. *Diagn. Interv. Radiol*. [Epub ahead of print]
2. Feng, Q.Z., Wang, J., and Sun, H. (2009) A sewing needle in liver: a case report and review of the literature. *Cases J*. **2,** 6520.
3. Ariyuca, S., Doğan, M., Kaya, A., and Ay, M. (2009) An unusual cause of liver abscess. *Liver Int.* **29,** 1552.

**This article should be cited as follows:**

Jutte, E.H. and Cense, H.A. (2010) Liver abscess due to sewing needle perforation. *TheScientificWorldJOURNAL* **10**, 1532–1534. DOI 10.1100/tsw.2010.142.
